# The survival benefit associated with complete macroscopic resection in epithelial ovarian cancer is histotype specific

**DOI:** 10.1093/jncics/pkae049

**Published:** 2024-06-20

**Authors:** Joanna M Porter, Iona McFarlane, Clare Bartos, Michael Churchman, James May, C Simon Herrington, Kathryn C Connolly, Neil A J Ryan, Robert L Hollis

**Affiliations:** The Nicola Murray Centre for Ovarian Cancer Research, Cancer Research UK Scotland Centre, Institute of Genetics and Cancer, University of Edinburgh, Edinburgh, UK; The Nicola Murray Centre for Ovarian Cancer Research, Cancer Research UK Scotland Centre, Institute of Genetics and Cancer, University of Edinburgh, Edinburgh, UK; The Nicola Murray Centre for Ovarian Cancer Research, Cancer Research UK Scotland Centre, Institute of Genetics and Cancer, University of Edinburgh, Edinburgh, UK; The Nicola Murray Centre for Ovarian Cancer Research, Cancer Research UK Scotland Centre, Institute of Genetics and Cancer, University of Edinburgh, Edinburgh, UK; The Royal Infirmary of Edinburgh, NHS Lothian, Edinburgh, UK; The Nicola Murray Centre for Ovarian Cancer Research, Cancer Research UK Scotland Centre, Institute of Genetics and Cancer, University of Edinburgh, Edinburgh, UK; The Royal Infirmary of Edinburgh, NHS Lothian, Edinburgh, UK; Edinburgh Cancer Centre, Western General Hospital, NHS Lothian, Edinburgh, UK; The Royal Infirmary of Edinburgh, NHS Lothian, Edinburgh, UK; The Nicola Murray Centre for Ovarian Cancer Research, Cancer Research UK Scotland Centre, Institute of Genetics and Cancer, University of Edinburgh, Edinburgh, UK

## Abstract

**Background:**

Complete macroscopic resection is a key factor associated with prolonged survival in ovarian cancer. However, most evidence derives from high-grade serous ovarian carcinoma, and the benefit of complete macroscopic resection in other histotypes is poorly characterized. We sought to determine which histotypes derive the greatest benefit from complete macroscopic resection to better inform future decisions on radical cytoreductive efforts.

**Methods:**

We performed multivariable analysis of disease-specific survival across 2 independent patient cohorts to determine the magnitude of benefit associated with complete macroscopic resection within each histotype.

**Results:**

Across both cohorts (Scottish: n = 1622; Surveillance, Epidemiology, and End Results [SEER]: n = 18 947), complete macroscopic resection was associated with prolonged disease-specific survival; this was more marked in the Scottish cohort (multivariable hazard ratio [HR] = 0.44, 95% confidence interval [CI] = 0.37 to 0.52 vs HR = 0.59, 95% CI = 0.57 to 0.62 in SEER). In both cohorts, clear cell ovarian carcinoma was among the histotypes to benefit most from complete macroscopic resection (multivariable HR = 0.23 and HR = 0.50 in Scottish and SEER cohorts, respectively); high-grade serous ovarian carcinoma patients demonstrated highly statistically significant and clinically meaningful survival benefit, but this was of lower magnitude than in clear cell ovarian carcinoma and endometrioid ovarian carcinoma across both cohorts. The benefit derived in low-grade serous ovarian carcinoma is also high (multivariable HR = 0.27 in Scottish cohort). Complete macroscopic resection was associated with prolonged survival in mucinous ovarian carcinoma patients in the SEER cohort (multivariable HR = 0.65), but the association failed to reach statistical significance in the Scottish cohort.

**Conclusions:**

The overall ovarian cancer patient population demonstrates clinically significant survival benefit associated with complete macroscopic resection; however, the magnitude of benefit differs between histotypes.

Ovarian cancer remains a major cause of morbidity and mortality in women; approximately 200 000 ovarian cancer deaths are reported each year worldwide (1). Epithelial ovarian cancer (ovarian carcinoma) is the most common form of the disease and comprises multiple histological types (histotypes): high-grade serous (70% of patients), endometrioid (10%), clear cell (10%), low-grade serous (≤5%), and mucinous (≤5%) (2-4). Ovarian carcinosarcomas—previously considered a separate disease entity—are now recognized to represent metaplastic carcinomas (4,5) and account for no more than 5% of diagnoses. A wealth of evidence now demonstrates that each histotype represents a unique disease entity, each with distinct developmental origins, molecular landscapes, intrinsic chemosensitivity, survival profile, and susceptibility to targeted molecular therapeutics (6-14).

The critical role of maximal cytoreductive surgery in improving ovarian carcinoma patient survival is well documented (3,15). Historically, achieving a maximal residual disease diameter of less than 2 cm was considered successful cytoreductive surgery (16). However, achievement of lower volume residual disease diameter (<1 cm, <0.5 cm) is associated with a greater survival advantage (17-19), and we now recognize that achieving complete macroscopic resection (also known as zero residual disease or R0) confers the most marked survival benefit (15,17,19,20).

While the importance of optimal cytoreduction is well established, the majority of data derive from high-grade serous ovarian carcinoma and have been extrapolated to the other histotypes. However, generalizing these data to other histotypes is problematic given that we now understand each histotype to represent a distinct disease entity (3,9,12,16,21-25). Therefore, it is not known if other histotypes benefit from complete macroscopic resection to the same extent as high-grade serous ovarian carcinoma. The magnitude of survival benefit derived from complete macroscopic resection may be modulated by factors such as the baseline chemosensitivity profile of each histotype and the intrinsic aggressiveness of each of these diseases. This is important because in many instances radical surgery is required to achieve complete macroscopic resection, which is associated with substantial morbidity and a recognized degree of mortality (26). Therefore, if women with non–high-grade serous ovarian carcinoma histotypes do not gain a meaningful survival benefit from surgery leading to complete macroscopic resection, the potential harms of such radical surgery may not be justified.

Here, we seek to determine the relative impact of complete macroscopic resection on survival across the ovarian carcinoma histotypes to improve our understanding of factors that influence patient outcomes, and to highlight patient groups for which the most aggressive surgical approaches are warranted.

## Methods

### Scottish ovarian cancer patient cohort

**Figure 1.**
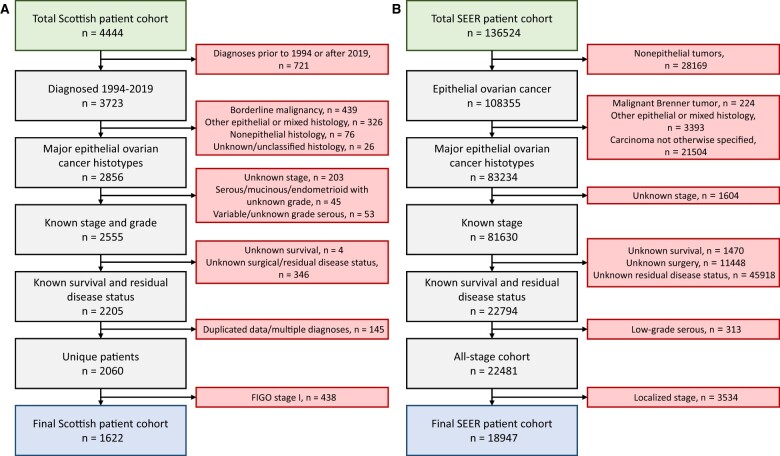
Flow diagrams for patient inclusion. **A)** Flow diagram for identifying the Scottish ovarian cancer patient cohort from the Edinburgh Ovarian Cancer database. **B)** Flow diagram for identifying the ovarian cancer patient cohort from the Surveillance, Epidemiology, and End Results database. FIGO = International Federation of Gynecology and Obstetrics; SEER = Surveillance, Epidemiology, and End Results.

A cohort of tubo-ovarian cancer patients (ovarian, primary peritoneal, or fallopian tube cancer) from Scotland was identified using the Edinburgh Ovarian Cancer Database, wherein the diagnosis, treatment, and outcome data for all women treated with histopathologically-confirmed ovarian cancer at the Edinburgh Cancer Centre (tertiary oncology center for South-East Scotland, United Kingdom) are prospectively recorded as part of routine care (16). A total of 4444 ovarian cancer patients were recorded with a diagnosis up to the end of December 2021. Of these, 2856 were high-grade serous ovarian carcinoma, low-grade serous ovarian carcinoma, endometrioid ovarian carcinoma, clear cell ovarian carcinoma, mucinous ovarian carcinoma, or ovarian carcinosarcoma and diagnosed January 1994 to December 2019, forming the basis of the study cohort (Supplementary Methods 1, available online) (see Figure 1, A). Patients of unknown International Federation of Gynecology and Obstetrics (FIGO) stage at diagnosis, unknown grade (where applicable), or unknown survival were excluded (n = 301). Of the remaining 2555 patients, 2205 received first-line cytoreductive surgery (primary cytoreduction or interval debulking following neoadjuvant chemotherapy) with known residual disease status; remaining patients were screened to exclude duplicated cases and patients with multiple primary diagnoses (n = 145), leaving a cohort of 2060 patients with FIGO stage I-IV disease at diagnosis (see Figure 1, A). Finally, stage I cases (n = 438) were removed, with 1622 patients remaining in the final study cohort.

High-grade serous ovarian carcinoma patients were divided into those who received primary debulking surgery vs those who received neoadjuvant chemotherapy followed by interval debulking surgery. For other histotypes, too few patients received interval debulking surgery to permit corresponding neoadjuvant-specific analysis. Of the 1622 patients in the Scottish study cohort, 69% had either undergone contemporary pathology review as part of recent molecular profiling studies (11,12,27-34) or represented recent diagnoses (2010 onward).

### Ethical approval

The study received institutional review board approval from the South East Scotland Cancer Information Research Governance Committee (Caldicott guardian approval CG/DF/E164, study reference CIR21087). For all participants, informed consent was obtained or was waived by the ethics committee due to the retrospective nature of this study. The study complied with all relevant ethical regulations and was performed in accordance with the declaration of Helsinki.

### Survival, Epidemiology, and End Results (SEER) database cohort

A validation cohort of patients from the SEER database was used to construct a corresponding validation dataset of 18 947 patients (Supplementary Methods 2, available online) (see Figure 1, B; Supplementary Table 1, available online).

### Statistical analysis

Survival time was calculated from date of pathologically-confirmed diagnosis to death from ovarian cancer (disease-specific survival); patients with other causes of death were censored. Cox proportional hazards regression models were used to determine differences in outcome, visualized using the Kaplan–Meier method. Multivariable analysis accounted for patient age, FIGO stage at diagnosis, and diagnosis period (5-year intervals). Differences in survival were presented as hazard ratios (HRs) and corresponding 95% confidence intervals (CIs). Median follow-up time was calculated using the reverse Kaplan–Meier method. Comparisons of frequency were made using the χ^2^ test. A calculated *P* value of less than .05 was considered statistically significant, while a calculated hazard ratio of less than 0.85 or greater than 1.15 was considered a potentially clinically meaningful effect size. Statistical tests were 2-sided.

Analyses were performed using R version 4.2.2 within R Studio version 2022.12.0 + 353, utilizing the following packages: finalfit, ggfortify, ggplot2, ggpubr, ggsurvfit, lifecycle, survival, survivalAnalysis, survminer, survMisc, tab, and table 1.

**Table 1.** Characteristics of ovarian carcinoma patient cohort from the Edinburgh Ovarian Cancer Database

Characteristics		Overall (n = 1622)		High-grade serous ovarian carcinoma (n = 1207)		Endometrioid ovarian carcinoma (n = 139)		Clear cell ovarian carcinoma (n = 104)		Low-grade serous ovarian carcinoma (n = 65)		Mucinous ovarian carcinoma (n = 38)		Ovarian carcinosarcoma (n = 69)	
		No.	(%)	No.	(%)	No.	(%)	No.	(%)	No.	(%)	No.	(%)	No.	(%)
Year of diagnosis	Pre-2005	722	44.5	529	43.8	78	56.1	36	34.6	25	38.5	16	42.1	38	55.1
	2005-2009	299	18.4	219	18.1	30	21.6	24	23.1	9	13.8	10	26.3	7	10.1
	2010-2014	317	19.5	222	18.4	23	16.5	33	31.7	14	21.5	11	28.9	14	20.3
	2015 onward	284	17.5	237	19.6	8	5.8	11	10.6	17	26.2	1	2.6	10	14.5
Age at diagnosis, years	Median (range)	64	56-71	64	57-72	61	53-69	62	53-69	60	43-68	55.5	48-65	67	61-73
International Federation of Gynecology and Obstetrics stage at diagnosis	II	246	15.2	92	7.6	65	46.8	53	51.0	11	16.9	17	44.7	8	11.6
	III	1044	64.4	836	69.3	57	41.0	38	36.5	45	69.2	19	50.0	49	71.0
	IV	332	20.5	279	23.1	17	12.2	13	10.6	9	13.8	2	5.3	12	17.4
Grade	Low grade or well differentiated or grade 1	—^a^		—		37	26.6	—		—		17	44.7	—	
	Moderately differentiated or grade 2	—		—		26	18.7	—		—		15	39.5	—	
	High grade or poorly differentiated or grade 3	—		—		76	54.7	—		—		6	15.8	—	
Neoadjuvant chemotherapy	Yes	290	17.9	266	22.0	7	5.0	6	5.8	7	10.8	0	0	4	5.8
	No	1332	82.1	941	78.0	132	95.0	98	94.2	58	89.2	38	100	65	94.2
Residual disease status	Complete macroscopic resection	574	35.4	358	29.7	74	53.2	64	61.5	29	44.6	23	60.5	26	37.7
	Macroscopic residual disease, <2 cm	362	22.3	299	24.8	17	12.2	13	12.5	16	24.6	1	2.6	16	23.2
	Macroscopic residual disease, ≥2 cm	624	38.5	506	41.9	39	28.1	24	23.1	19	29.2	13	34.2	23	33.3
	Macroscopic residual disease, unknown size	62	3.8	44	3.6	9	6.5	3	2.9	1	1.5	1	2.6	4	5.8
Vital status	Alive at last follow-up	334	20.6	221	18.3	53	38.1	23	22.1	24	36.9	11	28.9	2	2.9
	Deceased, ovarian cancer	1118	68.9	880	72.9	73	52.5	67	64.4	27	41.5	21	55.3	50	72.5
	Deceased, other causes	170	10.5	106	8.8	13	9.4	14	13.5	14	21.5	6	15.8	17	24.6
Follow-up	Median days	3298		3129		4788		2810		3439		2991		Not reached	

a Em dash denotes not applicable.

## Results

### Cohort characteristics

The Scottish study cohort comprised 1622 ovarian cancer patients with a diagnosis of FIGO stage II-IV high-grade serous ovarian carcinoma, low-grade serous ovarian carcinoma, endometrioid ovarian carcinoma, clear cell ovarian carcinoma, mucinous ovarian carcinoma, or ovarian carcinosarcoma between 1994 and 2019 (Figure 1, A). All patients underwent attempted cytoreductive surgery as part of first-line management (primary or interval debulking) and had known residual disease status (macroscopic residual disease vs complete macroscopic resection). Characteristics of the study cohort are summarized in Table 1. The median follow-up time for the study cohort was 108 months (Table 1).

**Figure 2.**
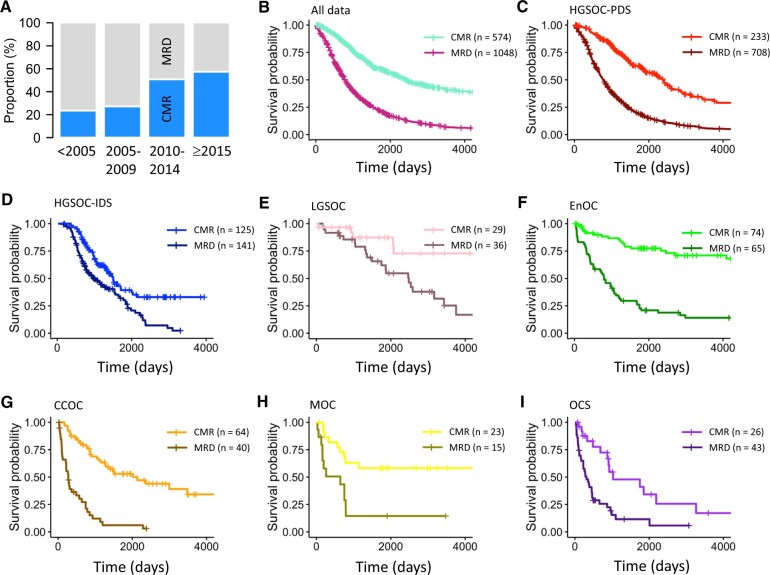
Impact of achieving complete macroscopic resection vs macroscopic residual disease in Scottish ovarian carcinoma cohort. **A)** Increase in frequency of achieving complete macroscopic resection over time. **B)** Overall impact of achieving complete macroscopic resection on disease-specific survival across the combined Scottish study cohort. **C)** Impact of complete macroscopic resection in high-grade serous ovarian carcinoma patients treated with primary debulking surgery. **D)** Impact of complete macroscopic resection in high-grade serous ovarian carcinoma patients treated with neoadjuvant chemotherapy and interval debulking surgery. **E)** Impact of complete macroscopic resection in low-grade serous ovarian carcinoma patients. **F)** Impact of complete macroscopic resection in endometrioid ovarian carcinoma patients. **G)** Impact of complete macroscopic resection in clear cell ovarian carcinoma patients. **H)** Impact of complete macroscopic resection in mucinous ovarian carcinoma patients. **I)** Impact of complete macroscopic resection in ovarian carcinosarcoma patients. CCOC = clear cell ovarian carcinoma; CMR = complete macroscopic resection; EnOC = endometrioid ovarian carcinoma; HGSOC = high-grade serous ovarian carcinoma; IDS = interval debulking surgery; LGSOC = low-grade serous ovarian carcinoma; MOC = mucinous ovarian carcinoma; MRD = macroscopic residual disease; OCS = ovarian carcinosarcoma; PDS = primary debulking surgery.

Of the Scottish cohort, 246 (15.2%), 1044 (64.4%), and 332 (20.5%) were of FIGO stage II, III and IV at diagnosis, respectively. A total of 574 patients had complete macroscopic resection after first-line cytoreductive surgery (35.4%). Of those with macroscopic residual disease, 624 (59.5%) had gross residual disease (≥2 cm), 362 (34.5%) had residual disease less than 2 cm in maximal diameter, and 62 (5.9%) had macroscopic residual disease of unknown size. There was a statistically significant increase in the frequency of achieving complete macroscopic resection over time (23.5% in pre-2005 to 57.4% in 2015-2019; *P* < 2.2e^-^^16^) (Figure 2, A).

**Figure 3.**
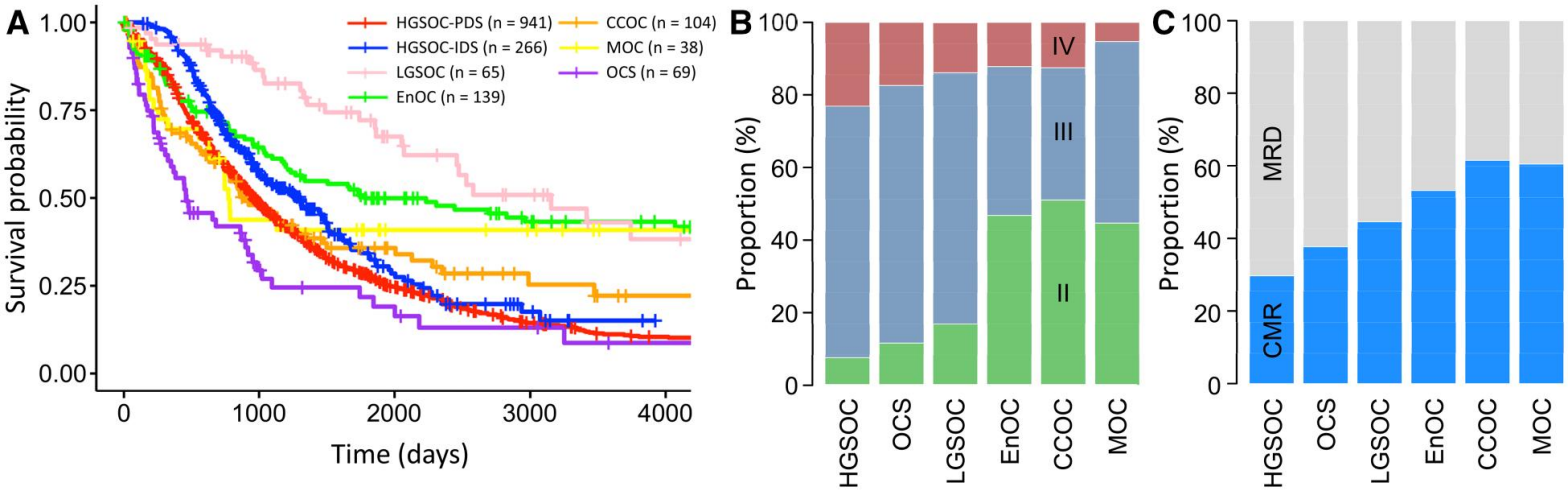
Features of ovarian carcinoma histotypes in the Scottish cohort. **A)** Disease-specific survival profile of each histotype. **B)** International Federation of Gynecology and Obstetrics stage at diagnosis across histotypes. **C)** Proportion of patients achieving complete macroscopic resection across each histotype. CCOC = clear cell ovarian carcinoma; CMR = complete macroscopic resection; EnOC = endometrioid ovarian carcinoma; HGSOC = high-grade serous ovarian carcinoma; IDS = interval debulking surgery; LGSOC = low-grade serous ovarian carcinoma; MOC = mucinous ovarian carcinoma; OCS = ovarian carcinosarcoma; MRD = macroscopic residual disease; PDS = primary debulking surgery.

Of the Scottish cohort, 1207 (74.4%), 139 (8.6%), 104 (6.4%), 65 (4.0%), 38 (2.3%), and 69 (4.3%) were high-grade serous ovarian carcinoma, endometrioid ovarian carcinoma, clear cell ovarian carcinoma, low-grade serous ovarian carcinoma, mucinous ovarian carcinoma, and ovarian carcinosarcoma, respectively. For analysis, high-grade serous ovarian carcinoma patients were divided into those who received primary debulking surgery (n = 941) vs neoadjuvant chemotherapy and interval debulking surgery (n = 266). Across the histotypes, ovarian carcinosarcoma patients demonstrated the poorest outcome, while low-grade serous ovarian carcinoma was associated with the most favorable survival (Figure 3, A).

### Univariable analysis of residual disease status

Univariable survival analysis of all patients according to residual disease status showed a substantial survival benefit associated with complete macroscopic resection compared with those with macroscopic residual disease (HR = 0.32, 95% CI = 0.28 to 0.37; P < 2e^−16^) (Figure 2, B).

Univariable analysis suggested achieving complete macroscopic resection was associated with varying degrees of statistically significant survival benefit across histotypes (complete macroscopic resection vs macroscopic residual disease in mucinous ovarian carcinoma: HR = 0.29, 95% CI = 0.12 to 0.71; clear cell ovarian carcinoma: HR = 0.19, 95% CI = 0.11 to 0.31; endometrioid ovarian carcinoma: HR = 0.19, 95% CI = 0.11 to 0.33; low-grade serous ovarian carcinoma: HR = 0.26, 95% CI = 0.10 to 0.69; ovarian carcinosarcoma: HR = 0.33, 95% CI = 0.17 to 0.62; high-grade serous ovarian carcinoma treated with primary debulking surgery: HR = 0.32, 95% CI = 0.26 to 0.39; high-grade serous ovarian carcinoma treated with interval debulking surgery: HR = 0.52, 95% CI = 0.38 to 0.72) (Figure 2, C-I). However, each histotype was associated with different distributions of stage at diagnosis (Figure 3, B), with corresponding differences in frequency of achieving complete macroscopic resection (Figure 3, C), highlighting the need for multivariable analysis.

### Multivariable analysis

Multivariable analysis confirmed statistically significant associations of stage at diagnosis, histotype, residual disease status, and period of diagnosis with survival time across the cohort (Supplementary Figure 1, A, available online). Advanced stage at diagnosis (FIGO IV vs II: HR = 3.47, 95% CI = 2.65 to 4.55; P < 2e^−16^), clear cell ovarian carcinoma (HR = 2.48 vs high-grade serous ovarian carcinoma treated with primary debulking surgery, 95% CI = 1.89 to 3.26; *P* = 6.91e^−11^), and ovarian carcinosarcoma (HR = 2.02 vs high-grade serous ovarian carcinoma treated with primary debulking surgery, 95% CI = 1.51 to 2.70; *P* = 2.18e^-^^6^) were associated with significantly poorer survival, while low-grade serous ovarian carcinoma (HR = 0.42 vs high-grade serous ovarian carcinoma treated with primary debulking surgery, 95% CI = 0.29 to 0.62; *P* = 1.26e^−5^) and achieving complete macroscopic resection were associated with statistically significantly prolonged survival (HR = 0.44 vs macroscopic residual disease, 95% CI = 0.37 to 0.52; P < 2e^−16^).

Histotype-specific analysis revealed a spectrum of survival benefit associated with complete macroscopic resection (Supplementary Figure 1, B-H, available online). Clear cell ovarian carcinoma patients derived the greatest survival benefit associated with complete macroscopic resection (HR = 0.23, 95% CI = 0.13 to 0.42; *P* = 1.20e^−6^) (Supplementary Figure 1, F, available online). The benefit in mucinous ovarian carcinoma failed to reach statistical significance (HR = 0.33, 95% CI = 0.09 to 1.18; *P* = .088) (Supplementary Figure 1, G, available online). The other histotypes demonstrated a gradient of statistically significant survival benefit associated with complete macroscopic resection (low-grade serous ovarian carcinoma: HR = 0.27, 95% CI = 0.09 to 0.77; endometrioid ovarian carcinoma: HR = 0.39, 95% CI = 0.22 to 0.70; ovarian carcinosarcoma: HR = 0.45, 95% CI = 0.21 to 0.96; high-grade serous ovarian carcinoma treated with primary debulking surgery: HR = 0.47, 95% CI = 0.38 to 0.60; high-grade serous ovarian carcinoma treated with interval debulking surgery: HR = 0.57, 95% CI = 0.41 to 0.80). Similar associations were identified upon analysis of overall (all-cause) survival (Supplementary Table 2, available online).

### Advanced stage disease

**Figure 4.**
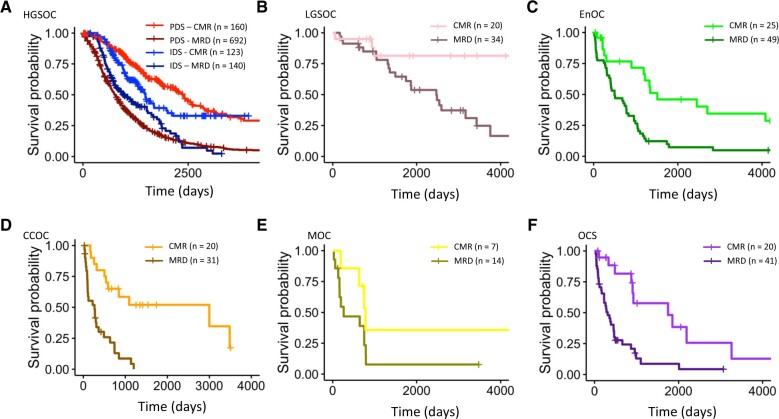
Impact of achieving complete macroscopic resection on disease-specific survival specifically in patients presenting with advanced stage disease at diagnosis (International Federation of Gynecology and Obstetrics stage III-IV) in the Scottish cohort. **A)** Impact of complete macroscopic resection in advanced stage high-grade serous ovarian carcinoma, stratified into those who received primary debulking surgery vs interval debulking surgery. **B)** Impact of complete macroscopic resection in advanced stage low-grade serous ovarian carcinoma patients. **C)** Impact of complete macroscopic resection in advanced stage endometrioid ovarian carcinoma patients. **D)** Impact of complete macroscopic resection in advanced stage clear cell ovarian carcinoma patients. **E)** Impact of complete macroscopic resection in advanced stage mucinous ovarian carcinoma patients. **F)** Impact of complete macroscopic resection in advanced stage ovarian carcinosarcoma patients. CCOC = clear cell ovarian carcinoma; CMR = complete macroscopic resection; EnOC = endometrioid ovarian carcinoma; HGSOC = high-grade serous ovarian carcinoma; IDS = interval debulking surgery; LGSOC = low-grade serous ovarian carcinoma; MOC = mucinous ovarian carcinoma; MRD = macroscopic residual disease; OCS = ovarian carcinosarcoma; PDS = primary debulking surgery.

Within advanced stage disease specifically (FIGO III-IV at diagnosis), low-grade serous ovarian carcinoma and clear cell ovarian carcinoma derived the greatest benefit from achieving complete macroscopic resection upon multivariable analysis (low-grade serous ovarian carcinoma: HR = 0.17, 95% CI = 0.05 to 0.64; clear cell ovarian carcinoma: HR = 0.19, 95% CI = 0.08 to 0.47) (Figure 4). Conversely, the survival benefit associated with complete macroscopic resection within advanced stage mucinous ovarian carcinoma patients was not statistically significant (HR = 0.32, 95% CI = 0.08 to 1.25; *P* = .102), though power was limited (Figure 4, E). The other histotypes demonstrated a gradient of statistically significant survival benefit associated with complete macroscopic resection upon multivariable analysis (ovarian carcinosarcoma: HR = 0.34, 95% CI = 0.14 to 0.78; endometrioid ovarian carcinoma: HR = 0.44, 95% CI = 0.23 to 0.85; high-grade serous ovarian carcinoma treated with primary debulking surgery: HR = 0.49, 95% CI = 0.39 to 0.63; high-grade serous ovarian carcinoma treated with interval debulking surgery: HR = 0.57, 95% CI = 0.41 to 0.80).

### Validation within the SEER database

**Figure 5.**
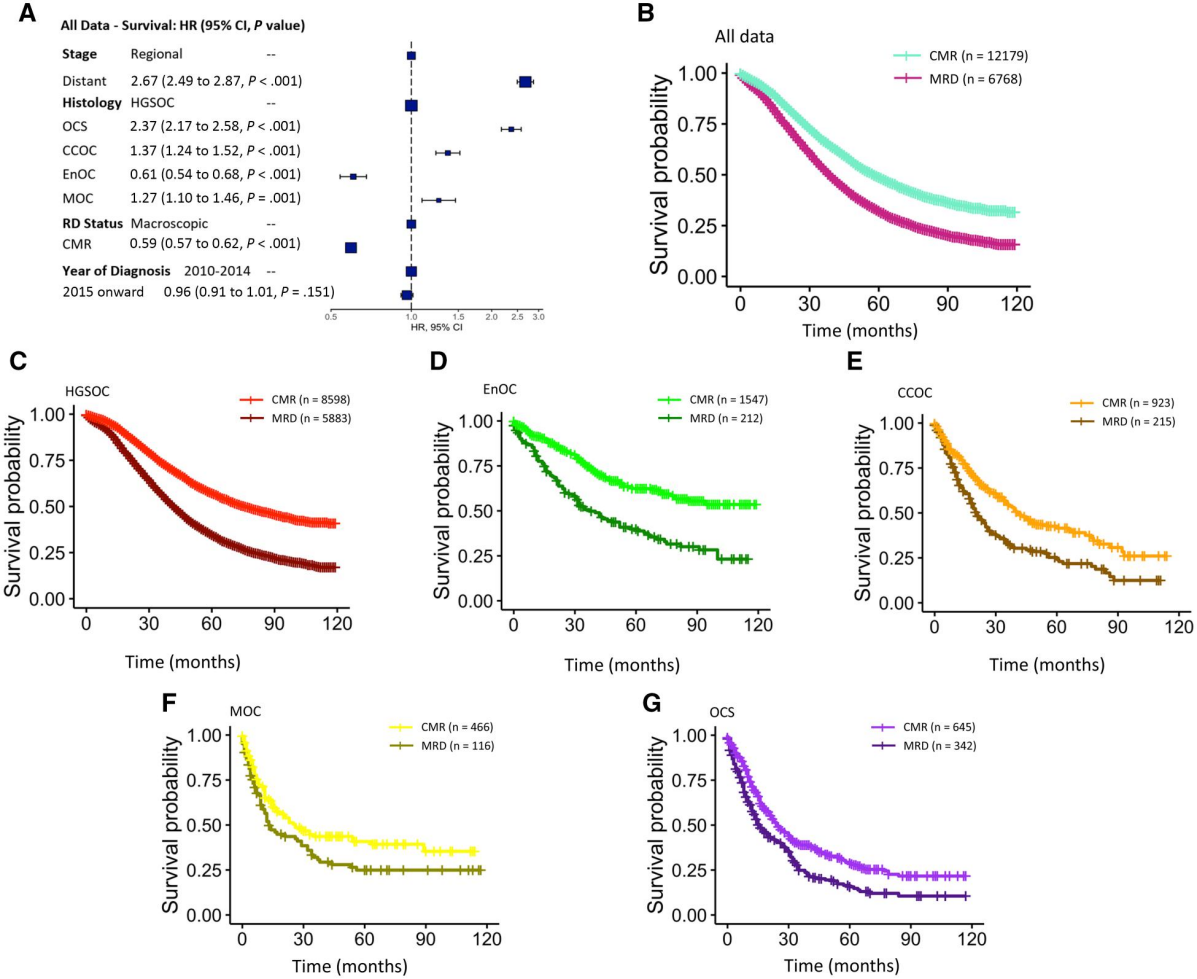
Impact of achieving complete macroscopic resection in the Surveillance, Epidemiology, and End Results (SEER) cohort. **A)** Overall multivariable forest plot of disease-specific survival analysis. **B)** Impact of achieving complete macroscopic resection across the overall SEER dataset. **C)** Impact of complete macroscopic resection in high-grade serous ovarian carcinoma patients. **D)** Impact of complete macroscopic resection in endometrioid ovarian carcinoma patients. **E)** Impact of complete macroscopic resection in clear cell ovarian carcinoma patients. **F)** Impact of complete macroscopic resection in mucinous carcinoma cancer patients. **G)** Impact of complete macroscopic resection in ovarian carcinosarcoma patients. CI = confidence interval; CCOC = clear cell ovarian carcinoma; CMR = complete macroscopic resection; EnOC = endometrioid ovarian carcinoma; HGSOC = high-grade serous ovarian carcinoma; HR = hazard ratio; MOC = mucinous ovarian carcinoma; MRD = macroscopic residual disease; OCS = ovarian carcinosarcoma; RD = residual disease.

A second cohort of 18 947 ovarian carcinoma patients (diagnosed 2010-2019) were extracted from the SEER database (Figure 1, B, and Table 2). The median follow-up time for the SEER cohort was 63 months; low-grade serous ovarian carcinoma patients were excluded because of low numbers and limited follow-up (Figure 1, B). Within the SEER cohort, complete macroscopic resection was associated with statistically significantly longer survival time (multivariable HR = 0.59, 95% CI = 0.57 to 0.62) (Figure 5, A).

**Table 2.** Characteristics of the Surveillance, Epidemiology, and End Results patient cohort

Surveillance, Epidemiology, and End Results cohort		Overall (n = 18 947)		High-grade serous ovarian carcinoma (n = 14 481)		Endometrioid ovarian carcinoma (n = 1759)		Clear cell ovarian carcinoma (n = 1138)		Mucinous ovarian carcinoma (n = 582)		Ovarian carcinosarcoma (n = 987)	
		No.	(%)	No.	(%)	No.	(%)	No.	(%)	No.	(%)	No.	(%)
Year of diagnosis	2010-2014	10 387	54.8	7810	53.9	1086	61.7	631	55.4	343	58.9	517	52.4
	2015-2019	8560	45.2	6671	46.1	673	38.3	507	44.6	239	41.1	470	47.6
Age at diagnosis, years	Median age category	60-64		60-64		55-59		55-59		55-59		65-69	
SEER stage at diagnosis	Regional	5245	27.7	2691	18.6	1260	71.6	700	61.5	338	58.1	256	25.9
	Distant	13 702	72.3	11 790	81.4	499	28.4	438	38.5	244	41.9	731	74.1
Residual disease status	Complete macroscopic resection	12 179	64.3	8598	59.4	1547	87.9	923	81.1	466	80.1	645	65.3
	Macroscopic residual disease	6768	35.7	5883	40.6	212	12.1	215	18.9	116	19.9	342	34.7
Vital status	Alive at last follow-up	10 009	52.8	7318	50.5	1337	76.0	673	59.1	340	58.4	341	34.5
	Deceased, ovarian cancer	7932	41.9	6412	44.3	339	19.3	408	35.9	195	33.5	578	58.6
	Deceased, other causes	1006	5.3	751	5.2	83	4.7	57	5.0	47	8.1	68	6.9
Follow-up	Median months	63		62		69		63		65		60	

In histotype-specific multivariable analysis, endometrioid ovarian carcinoma and clear cell ovarian carcinoma patients derived the greatest survival benefit from achieving complete macroscopic resection (HR = 0.41, 95% CI = 0.31 to 0.53 and HR = 0.50, 95% CI = 0.39 to 0.63, respectively). High-grade serous ovarian carcinoma (HR = 0.61, 95% CI = 0.58 to 0.64), mucinous ovarian carcinoma (HR = 0.65, 95% CI = 0.46 to 0.91), and ovarian carcinosarcoma patients (HR = 0.66, 95% CI = 0.56 to 0.79) demonstrated statistically significantly prolonged survival associated with achieving complete macroscopic resection (Figure 5).

Multivariable histotype-specific analysis of SEER patients with distant stage disease demonstrated that endometrioid ovarian carcinoma and clear cell ovarian carcinoma cases derived the greatest survival benefit from complete macroscopic resection (HR = 0.42, 95% CI = 0.31 to 0.56; HR = 0.55, 95% CI = 0.42 to 0.72, respectively). Mucinous ovarian carcinoma, high-grade serous ovarian carcinoma, and ovarian carcinosarcoma patients also derived statistically significant benefit but of lower magnitude (HR = 0.63, 95% CI = 0.44 to 0.91; HR = 0.63, 95% CI = 0.60 to 0.66; HR = 0.68, 95% CI = 0.57 to 0.82, respectively).

## Discussion

Over the last two decades, our understanding of ovarian carcinoma has advanced substantially. We now recognize the existence of multiple clinically and molecularly distinct ovarian cancer histotypes, each with distinct survival and treatment sensitivity profiles (2). Our knowledge of factors associated with patient outcome has also increased greatly, with achievement of complete macroscopic resection at first-line cytoreduction emerging as a key factor associated with improved survival (35). However, understanding of such factors has primarily been driven by the most common histotype, high-grade serous ovarian carcinoma, which has dominated studies to date (35). Accordingly, although the importance of optimal cytoreduction has become widely recognized, little is known regarding the relative survival benefit associated with complete macroscopic resection across different histotypes. We sought to improve our understanding of the survival advantage derived when complete macroscopic resection is achieved at first-line cytoreduction within each histotype. We used multivariable analysis to quantify the magnitude of survival benefit independent of other clinicopathological factors, as the distribution of these factors is known to vary across histotypes (2).

Using an ovarian carcinoma patient cohort from Scotland with rich clinical annotation, we show that clear cell ovarian carcinoma and low-grade serous ovarian carcinoma demonstrate the most marked survival benefit associated with complete macroscopic resection compared with other histotypes (multivariable HR = 0.23 and 0.27 compared with HR = 0.47 in high-grade serous ovarian carcinoma treated with primary debulking surgery). It is feasible that differences in intrinsic chemosensitivity may contribute to this difference; low-grade serous ovarian carcinoma and clear cell ovarian carcinoma demonstrate marked intrinsic chemoresistance (36,37), and macroscopic residual disease in these histotypes may lead to rapid progression even in the context of adjuvant chemotherapy. Conversely, in high-grade serous ovarian carcinoma, which is highly chemosensitive (38), residual lesions are more likely to respond to subsequent platinum-based adjuvant therapy, and the benefit from achieving complete macroscopic resection at surgery may therefore be less extreme. In keeping with this notion, ovarian carcinosarcoma, which demonstrates intermediate levels of intrinsic chemosensitivity (11), derives a clinically and statistically significant benefit that is lesser in magnitude vs low-grade serous ovarian carcinoma and clear cell ovarian carcinoma. However, endometrioid ovarian carcinoma, which demonstrates intermediate chemosensitivity (2), was among the histotypes to benefit most from complete macroscopic resection, suggesting factors beyond intrinsic chemosensitivity that modulate the benefit from complete macroscopic resection. Alongside chemoresistance, low-grade serous ovarian carcinoma is characterized by a more gradual disease progression course, with prolonged post-relapse survival compared with high-grade serous ovarian carcinoma (25). The distinct clinical behavior of low-grade serous ovarian carcinoma may also contribute toward the large impact on survival when achieving complete macroscopic resection; it is feasible that the dramatic reduction in cell numbers due to maximal cytoreduction may produce an extended subclinical disease course because of the lagging proliferation rate of low-grade serous ovarian carcinoma cells. It is also feasible that specific biological events modulate the survival benefit from complete macroscopic resection; within high-grade serous ovarian carcinoma, it has been suggested that the burden of tumor-infiltrating immune cells may impact the degree to which complete macroscopic resection improves survival (39). These data suggest that molecular subtypes within specific histotypes may also demonstrate differences in the survival benefit associated with complete macroscopic resection.

We identified a larger survival benefit associated with complete macroscopic resection in high-grade serous ovarian carcinoma patients who underwent primary debulking surgery compared with those who received neoadjuvant chemotherapy followed by interval debulking surgery (multivariable HR = 0.47 vs 0.57). It is possible that the timing of chemotherapy itself modulates the survival benefit derived from successful cytoreduction; induction of platinum resistance during neoadjuvant chemotherapy has been raised as a concern in the current era of increased neoadjuvant chemotherapy utilization (40). However, inherent differences in the interval debulking surgery population, rather than neoadjuvant chemotherapy itself, may well underlie this observation. Patients undergoing neoadjuvant chemotherapy typically have widely disseminated, unresectable disease or harbor comorbidities rendering them unsuitable for primary debulking surgery. Caution is therefore warranted in the interpretation of these findings.

A corresponding patient cohort from the SEER database confirmed clear cell ovarian carcinoma as one of the histotypes that benefits most from complete macroscopic resection. Limited follow-up time and smaller-than-expected numbers of low-grade serous ovarian carcinoma patients prevented validation of our findings on this histotype from the Scottish cohort within SEER. We were also unable to account for primary vs interval debulking surgery in the SEER cohort because of the less detailed treatment information available. In contrast to the Scottish cohort, the endometrioid ovarian carcinoma patient group within SEER demonstrated the largest degree of survival benefit associated with complete macroscopic resection, though the effect size in both studies was similar (multivariable HR = 0.41 in SEER vs HR = 0.39 in Scottish cohort), and endometrioid ovarian carcinoma was among the histotypes that benefited most from complete macroscopic resection across both cohorts. Mucinous ovarian carcinoma failed to demonstrate a statistically significant survival benefit associated with complete macroscopic resection in the Scottish cohort; in the SEER cohort, the benefit was statistically significant, though the effect size was still lower for mucinous ovarian carcinoma in this cohort than clear cell ovarian carcinoma (mucinous ovarian carcinoma, HR = 0.63 in SEER; clear cell ovarian carcinoma, HR = 0.50 in SEER). In contrast to the Scottish cohort, the ovarian carcinosarcoma group appeared to derive the least benefit from complete macroscopic resection. In both cohorts, high-grade serous ovarian carcinoma patients represented a patient group who demonstrated a lower magnitude of survival benefit compared with most other histotypes, though it remains clear that the degree of benefit is highly clinically and statistically meaningful. Some of these discrepancies may be due to intrinsic differences between our two study cohorts. In particular, the magnitude of survival benefit associated with complete macroscopic resection across the overall population in SEER was less marked than in the Scottish ovarian cancer cohort (multivariable HR = 0.59 in SEER vs HR = 0.44 in the Scottish cohort). Both cohorts identify clear cell ovarian carcinoma and endometrioid ovarian carcinoma as histotypes that are among those that derive the largest degree of survival benefit from complete macroscopic resection. These histotypes have a number of shared molecular features, and both are related to endometriosis (2); strong associations between complete macroscopic resection and improved survival represents an additional commonality between these tumor types (2).

A major strength of our study is the use of multiple geographically distinct cohorts of ovarian carcinoma patients from contrasting sources. The Scottish cohort represents a smaller group of richly annotated individuals from a single tertiary oncology center in the United Kingdom with a single data source specific to ovarian cancer, while the SEER database harbors a large number of patients, aggregated from centers across the United States into a pancancer database. Long follow-up time, recognition of histotypes as distinct disease entities, and multivariable analysis represent further strengths of this work. Lack of central pathology review is a weakness of our study, though 69% of the Scottish cohort had either undergone pathology review as part of previous studies or was contemporary diagnoses. We were unable to perform any pathology review within the SEER cohort, but we included only relatively recent diagnoses from this source (2010-2019). However, we cannot preclude the possibility of low-level histotype misclassification within both cohorts; in particular, a proportion of low-grade serous ovarian carcinoma patients in the Scottish cohort were historic diagnoses of serous grade 1 carcinomas. Moreover, the treatment paradigm in ovarian cancer has shifted substantially in recent years, particularly with the uptake of maintenance poly-(ADP-ribose) polymerase inhibitors (13,41); the bulk of our study populations were treated prior to the era of poly-(ADP-ribose) polymerase inhibitor maintenance therapy. In addition, other histotype-specific management strategies are now being adopted or investigated in ovarian cancer, including MEK inhibition for low-grade serous ovarian carcinoma and immunotherapy for clear cell ovarian carcinoma (2). Changes in ovarian cancer management strategies may impact the benefit from complete macroscopic resection across histotypes. Lastly, though our study cohorts represent relatively large populations, particularly of the less common histotypes, statistical power was still limited in some analyses, principally within the Scottish cohort.

Together, these data further underscore the pivotal role of maximal cytoreduction in ovarian cancer management and highlight patient groups most likely to derive the greatest benefit from radical and ultraradical surgical approaches. Clear cell ovarian carcinoma and low-grade serous ovarian carcinoma patients represent some of the groups who benefit most from achieving complete macroscopic resection; given that these cases are unlikely to respond well to neoadjuvant chemotherapy, complete primary cytoreduction is a major priority for these patients. Endometrioid ovarian carcinoma also represents a histotype in which complete macroscopic resection is associated with marked patient survival benefit. Engagement with multiple surgical teams to improve the likelihood of resecting disease at hepatobiliary, gastrointestinal, and other anatomical sites beyond the pelvis will be key for delivering optimal outcomes for such cases that present with extensive advanced-stage disease. More work is also required to understand the impact of resecting disease outside of the pelvis and abdomen.

Achievement of complete macroscopic resection across the overall ovarian cancer patient population is associated with markedly prolonged survival time, though differences in the degree of survival benefit are apparent across different histotypes. Patients with clear cell ovarian carcinoma derive one of the greatest survival benefits associated with complete macroscopic resection. High-grade serous ovarian carcinoma patients demonstrate highly clinically and statistically meaningful survival benefit, but the magnitude of benefit is lower than in some of the other histotypes. The extent of benefit from complete macroscopic resection does not appear to relate solely to levels of intrinsic chemosensitivity of each histotype.

## Supplementary Material

pkae049_Supplementary_Data

## Data Availability

Data from the SEER database is publicly available; users should register for access through the SEER program website (https://seer.cancer.gov/data-software/). Line-by-line data for the Scottish cohort cannot be made publicly available to comply with our local ethics framework. We are happy to support access to these data on a project-by-project basis via contact with the corresponding authors; this may require additional ethical approval and is subject to compliance with our ethical regulations.
