# Supplementary material for: The survival benefit associated with complete macroscopic resection in epithelial ovarian cancer is histotype specific
Source: JNCI Cancer Spectr. 2024 Jun 20;8(4):pkae049. doi: 10.1093/jncics/pkae049 (PMC11233146; doi:10.1093/jncics/pkae049)
Supplement: pkae049_Supplementary_Data [file pkae049_supplementary_data.docx]

**The survival benefit associated with complete macroscopic resection in epithelial ovarian cancer is histotype-specific**

**JM Porter, et al.**

**SUPPLEMENTARY METHODS 1: Histotype classification from Edinburgh Ovarian Cancer Database**

Cases of OCS, EnOC, CCOC and MOC histotypes were identified directly from the Edinburgh Ovarian Cancer Database. The HGSOC cohort comprised cases with a contemporary diagnosis of HGSOC, alongside historic cases documented as poorly differentiated/grade 3 and moderately differentiated/grade 2 serous carcinoma. The LGSOC group comprised contemporary diagnoses of LGSOC alongside well differentiated/grade I serous carcinomas.

**SUPPLEMENTARY METHODS 2: SEER cohort**

A validation cohort of patients from the Surveillance, Epidemiology, and End Results (SEER) database (November 2021 SEER submission: 2000-2019, 17 registries; selected for malignant behaviour and known age) were used to construct a validation dataset (Figure 1B). From 7,788,415 returned cancer diagnoses from the case listing session, 136,524 were identified as ovarian (C56.9), fallopian tube (C57.0) or peritoneal cancers (C48.0, C48.1, C48.2, C48.8) diagnosed between 2010-2019 (Figure 1B). To reduce potential contamination with misclassified histotypes, SEER cases were restricted to a more contemporary study period compared to the Scottish cohort, where pathology review was available for a substantial proportion of cases from recent molecular profiling studies (see main methods). Exclusion criteria were: non-epithelial tumours (n=28169), mixed carcinomas (n=304), carcinomas of unspecified histology (n=21504), malignant Brenner tumours (n=224), other histotypes (n=3089), unknown stage (n=1604), unknown survival/vital status (n=1470), no data on first-line surgery or no first-line surgery (n=11448), and unknown residual disease status after surgery (n=45918) (Figure 1B). LGSOC cases were excluded due to markedly short follow-up time and low numbers compared to other histotypes. Finally, cases with ‘localized’ disease (stage I) at diagnosis were excluded (n=_3534), leaving a study validation cohort of 18947 SEER cases. Morphology codes were mapped to histotypes as outlined in Supplementary table 1.

Supplementary table 1. Histotype classification scheme based on the International Classification of Diseases for Oncology*,* 3rd edition (ICD-O-3) morphology/behaviour codes and histology

| **Histology** | **Morphology/Behaviour Codes** |
| --- | --- |
| Serous | 8441/3: Serous cystadenocarcinoma, NOS |
|  | 8442/3: Proliferating serous carcinoma, malignant |
|  | 8460/3: Papillary serous cystadenocarcinoma |
|  | 8461/3: Serous surface papillary carcinoma |
|  | 8462/3: Papillary serous cystadenocarcinoma |
|  | 8463/3: Serous surface papillary carcinoma |
|  | 9014/3: Serous adenocarcinofibroma |
| Endometrioid | 8380/3: Endometrioid carcinoma |
|  | 8381/3: Endometrioid adenofibroma, malignant |
|  | 8382/3: Endometrioid adenocarcinoma, secretory variant |
|  | 8383/3: Endometrioid adenocarcinoma, ciliated cell variant |
|  | 8570/3: Adenocarcinoma with squamous metaplasia |
| Mucinous | 8470/3: Mucinous cystadenocarcinoma, NOS |
|  | 8471/3: Papillary mucinous cystadenocarcinoma |
|  | 8472/3: Mucinous cystadenocarcinoma |
|  | 8480/3: Mucinous adenocarcinoma |
|  | 8481/3: Mucin-producing adenocarcinoma |
| Clear cell | 8310/3: Clear cell adenocarcinoma, NOS |
|  | 8313/3: Clear cell adenocarcinofibroma |
|  | 8443/3: Clear cell cystadenocarcinoma |
|  | 8444/3: Clear cell cystic tumor, malignant |
| OCS | 8575/3: Metaplastic carcinoma, NOS |
|  | 8950/3: Mullerian mixed tumor |
|  | 8951/3: Mesodermal mixed tumor |
|  | 8980/3: Carcinosarcoma, NOS |
|  | 8981/3: Carcinosarcoma, embryonal |

Supplementary table 2. Impact of achieving complete macroscopic resection (CMR) on all-cause mortality (overall survival):

|  | **Scottish cohort** | | | **SEER cohort** | | |
| --- | --- | --- | --- | --- | --- | --- |
|  | **Multivariable HR for CMR** | **95% CI** | **P-value** | **Multivariable HR for CMR** | **95% CI** | **P-value** |
| All data | 0.47 | 0.41-0.55 | < 2e^-16^ | 0.62 | 0.60-0.65 | < 2e^-16^ |
| HGSOC-PDS | 0.51 | 0.41-0.63 | 4.00e^-10^ | 0.63 | 0.60-0.67 | < 2e^-16^ |
| HGSOC-IDS | 0.56 | 0.41-0.75 | 0.0002 |  |  |  |
| LGSOC | 0.35 | 0.16-0.79 | 0.012 | - | - | - |
| EnOC | 0.44 | 0.26-0.74 | 0.002 | 0.45 | 0.35-0.58 | 1.67e^-10^ |
| CCOC | 0.26 | 0.15-0.46 | 3.29e^-6^ | 0.53 | 0.42-0.67 | 4.22e^-8^ |
| MOC | 0.40 | 0.13-1.29 | 0.127 | 0.61 | 0.44-0.82 | 0.001 |
| OCS | 0.52 | 0.28-0.96 | 0.038 | 0.65 | 0.55-0.76 | 1.87e^-7^ |

HR, hazard ratio. HGSOC, high grade serous ovarian carcinoma. PDS, primary debulking surgery. IDS, interval debulking surgery. LGSOC, low grade serous ovarian carcinoma. EnOC, endometrioid ovarian carcinoma. CCOC, clear cell ovarian carcinoma. MOC, mucinous ovarian carcinoma. OCS, ovarian carcinosarcoma.


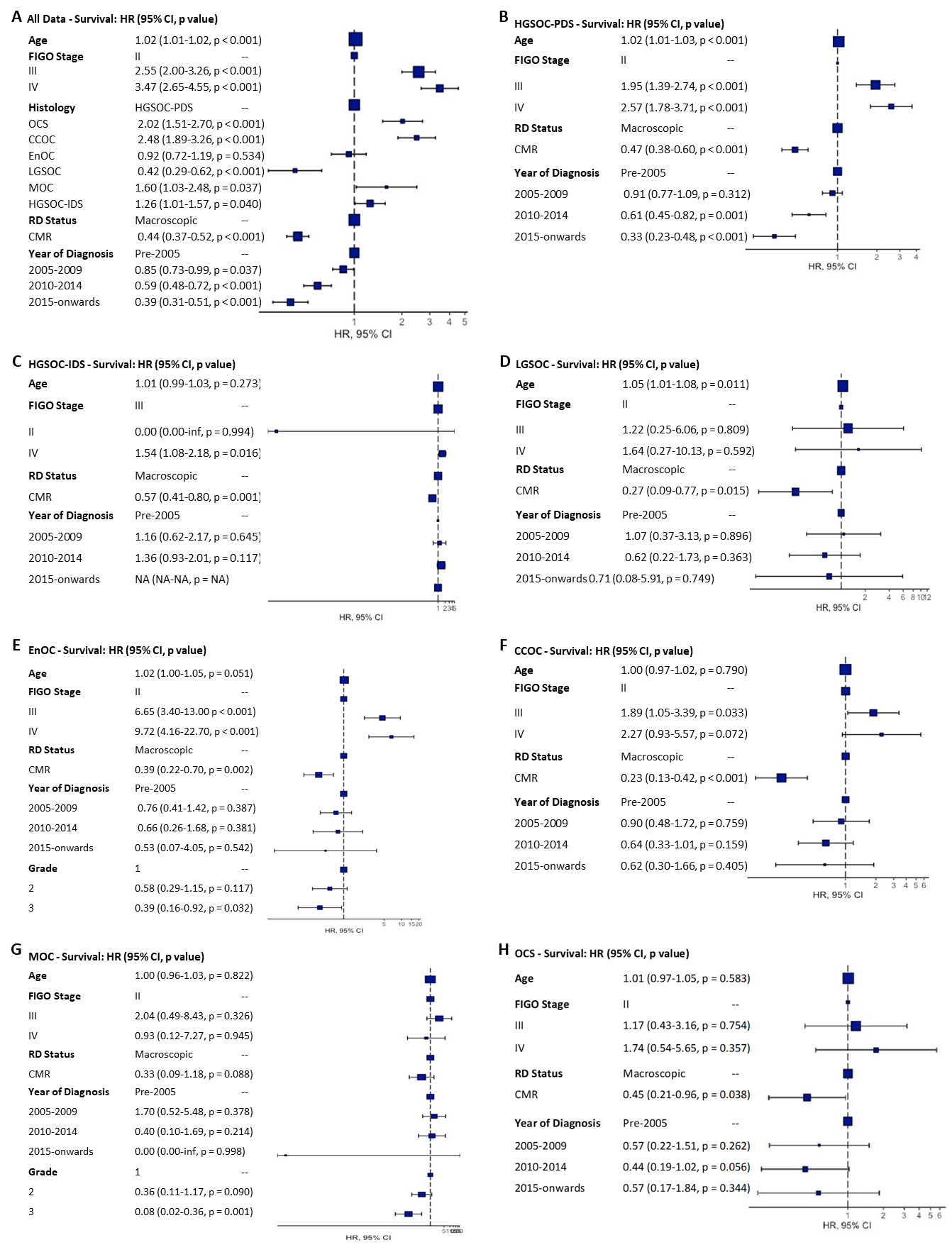


Supplementary Figure 1. Forest plots of multivariable disease-specific survival analysis in the Scottish ovarian carcinoma (OC) patient cohort. (A) Overall study cohort. (B) High grade serous OC (HGSOC) cases receiving primary debulking surgery (PDS). (C) HGSOC cases receiving interval debulking surgery (IDS). (D). Low grade serous OC (LGSOC) cases. (E) Endometrioid OC (EnOC) cases. (F) Clear cell OC (CCOC) cases. (G) Mucinous OC (MOC) cases. (H) Ovarian carcinosarcoma (OCS) cases.
